# Reply: Addressing the Overlooked: Orthostatic Intolerance Syndromes in Hematopoietic Stem Cell Transplant Recipients

**DOI:** 10.1016/j.jaccao.2023.12.007

**Published:** 2024-02-20

**Authors:** Alexi Vasbinder, Salim Hayek

**Affiliations:** a University of Michigan, Ann Arbor, Michigan, USA

We thank Dr Thomas and colleagues for their insightful letter regarding our recent publication on cardiovascular outcomes post-hematopoietic stem cell transplantation (HSCT).^1^ We appreciate their emphasis on orthostatic intolerance syndromes (OIS), particularly postural orthostatic tachycardia syndrome (POTS), as a potential complication following HSCT and other hyperinflammatory processes.

We concur that POTS, a form of dysautonomia, involves a disruption in the autonomic nervous system, leading to significant alterations in heart rate and blood pressure upon standing.^2^ Although cardiovascular specialists often address POTS due to its impact on the cardiovascular system, it is indeed crucial to recognize that POTS is fundamentally a dysautonomic condition. The underlying pathophysiology of POTS is complex and multifactorial, involving potential mechanisms such as peripheral denervation, abnormal blood volume regulation, and hyperadrenergic states.^3^^,^^4^

Given the underdiagnosis and wide-ranging severity of POTS, along with its diagnostic challenges and multifactorial nature, its systematic assessment as a cardiovascular outcome in our study was not feasible. Our focus was on direct cardiovascular outcomes post-HSCT, and although the significance of POTS is not understated, its classification as a primary cardiovascular event was beyond our study's scope.

However, we agree that more comprehensive research into OIS, including POTS, in the context of HSCT is warranted. Dr Thomas and colleagues’ letter underscores the necessity for a broader perspective in evaluating and documenting long-term complications following intensive treatments like HSCT. We hope that our study, complemented by their insights, will stimulate further research in this area, ultimately enhancing patient care and outcomes.
